# Near Real-Time Monitoring of Clinical Events Detected in Swine Herds in Northeastern Spain

**DOI:** 10.3389/fvets.2020.00068

**Published:** 2020-02-18

**Authors:** Ana Alba-Casals, Eduard Allue, Vicens Tarancon, Jordi Baliellas, Elena Novell, Sebastián Napp, Lorenzo Fraile

**Affiliations:** ^1^IRTA, Centre de Recerca en Sanitat Animal (CReSA, IRTA-UAB), Campus de la Universitat Autònoma de Barcelona, Barcelona, Spain; ^2^The OIE Collaborating Centre for the Research and Control of Emerging and Re-emerging Diseases in Europe (IRTA-CReSA), Barcelona, Spain; ^3^Grup de Sanejament Porcí, Lleida, Spain; ^4^Departament de Ciència Animal, ETSEA, Universitat de Lleida-Agrotecnio, Lleida, Spain

**Keywords:** swine health, endemic diseases, monitoring, data mining, web application, endemic-epidemic multivariate time-series model

## Abstract

Novel techniques of data mining and time series analyses allow the development of new methods to analyze information relating to the health status of the swine population in near real-time. A swine health monitoring system based on the reporting of clinical events detected at farm level has been in operation in Northeastern Spain since 2012. This initiative was supported by swine stakeholders and veterinary practitioners of the Catalonia, Aragon, and Navarra regions. The system aims to evidence the occurrence of endemic diseases in near real-time by gathering data from practitioners that visited swine farms in these regions. Practitioners volunteered to report data on clinical events detected during their visits using a web application. The system allowed collection, transfer and storage of data on different clinical signs, analysis, and modeling of the diverse clinical events detected, and provision of reproducible reports with updated results. The information enables the industry to quantify the occurrence of endemic diseases on swine farms, better recognize their spatiotemporal distribution, determine factors that influence their presence and take more efficient prevention and control measures at region, county, and farm level. This study assesses the functionality of this monitoring tool by evaluating the target population coverage, the spatiotemporal patterns of clinical signs and presumptive diagnoses reported by practitioners over more than 6 years, and describes the information provided by this system in near real-time. Between January 2012 and March 2018, the system achieved a coverage of 33 of the 62 existing counties in the three study regions. Twenty-five percent of the target swine population farms reported one or more clinical events to the system. During the study period 10,654 clinical events comprising 14,971 clinical signs from 1,693 farms were reported. The most frequent clinical signs detected in these farms were respiratory, followed by digestive, neurological, locomotor, reproductive, and dermatological signs. Respiratory disorders were mainly associated with microorganisms of the porcine respiratory disease complex. Digestive signs were mainly related to colibacilosis and clostridiosis, neurological signs to Glässer's disease and streptococcosis, reproductive signs to PRRS, locomotor to streptococcosis and Glässer's disease, and dermatological signs to exudative epidermitis.

## Introduction

The prevention and control of diseases are essential to ensure efficient and sustainable swine production. Getting updated information on the health status of the target swine population in near real-time can facilitate the implementation of efficient measures by swine stakeholders, veterinary practitioners, and government. Innovative surveillance methods based on the analyses of various types of data, which may serve as indirect health indicators, are under development (1–3). The ability to collect data in a cost-effective and timely manner from a wide range of sources, the use of data mining techniques and time series analyses, and the possibility of generating dynamic reproducible reports, has led to the development of new ways of conducting surveillance in near real-time (4, 5).

In recent years, the Spanish swine sector has grown significantly, with over 50% of the pig herds concentrated in Catalonia and Aragon (regions located in the North East of the country). In those areas, the number of sows in large-scale operations has increased, and an important proportion of facilities are part of integrated industries with highly specialized farrowing, post-weaning, and finishing sites (6). In this context of swine production, it is essential to maintain a good sanitary status.

The Porcine Sanitation Group of Lleida, Spain (GSP) is a non-profit association that brings together pig owners, independent breeders, and companies associated to the swine sector in Northeastern Spain. The GSP aims to improve the swine health in farms and collaborates closely with the official animal health authorities carrying out actions related to disease surveillance, prevention, and control. In 2012, the GSP decided to carry out a near-real time monitoring system in Aragon, Catalonia and Navarra to gather data on clinical events detected by practitioners. The GSP hypothesized that, by monitoring, targeting, and reporting clinical signs and presumptive diagnoses, it would be possible to reveal in near real-time the occurrence of endemic diseases that are not notifiable. This information might help assess the spatiotemporal distribution of such diseases in these populations, identify subpopulations at high risk and factors that influence disease presence. Practitioners and swine stakeholders would benefit from this information to plan and take more efficient control measures.

It is important to highlight that the initial intention of this monitoring tool was not associated with a pre-defined control plan against a specific disease. The main aim of the tool was to gather data from swine herds in near real-time and provide accessible and regularly updated information to veterinary practitioners and swine stakeholders. The system aimed to visually track the spatiotemporal distribution and spread of endemic diseases and support the decision of where and when actions were necessary. Moreover, the system aimed to enhance the communication and cooperation within the swine sector in Northeastern Spain. This work aims to evaluate the functionality of this system developed to monitor the frequency of endemic diseases in the swine population at region and county level, and discusses the advantages and limitations related to its implementation.

## Materials and Methods

To illustrate how the GSP monitoring system operates, we analyzed the data of clinical events reported voluntarily by veterinary clinicians from swine farms of the Catalonia, Aragon, and Navarra regions (Northeastern Spain) between January 2012 and March 2018.

### Development of a Web Application to Report Clinical Events Detected in Swine Farms

**Figure 1:**
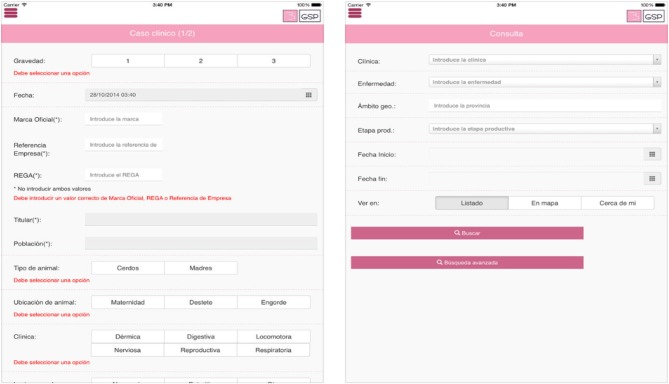
Interface of a web application to record clinical event data from swine herds in Northeastern Spain. Fields written in Spanish. English translation: Case (*Caso clínico*), Severity (*Gravedad*), Date (*Fecha*), Regional official farm identifier (*Marca Oficial*). Company reference (*Referencia empresa*), National official farm identifier (*REGA*), Owner (*Titular*), Municipality (*Población*), Type of animal (*Tipo de animal*), Piglets or fattening pigs (*Cerdos*), Sows (*Madres*), Farm Location of the affected animals within the farm (*Ubicación del animal*), Maternity (*Maternidad*), Nursery (*Destete*), Fattening (*Engorde*), Clinical signs (C*línica*), Dermatological (*Dermatológica*), Digestive (*Digestiva*), Locomotor (*Locomotora*), Neurological (*Nerviosa*), Reproductive (*Reproductiva*), Respiratory (*Respiratoria*), Presumptive disease (*Enfermedad*), Geographical situation (Á*mbito geográfico*), Productive phase (*Etapa productiva*), Date of clinical onset (*Fecha inicio*), Date of clinical end (*Fecha fin*), See (*Ver en*), List (*Listado*), Map representation (*En mapa*), Closest clinical cases (*Cerca de mí*), Search (*Buscar*), Advanced search (*Búsqueda avanzada*).

The researchers and technicians of GSP, in collaboration with many swine stakeholders and veterinary practitioners, developed a web application (app) to collect and store data on clinical events detected by veterinarians during their visits to farms. Before launching the system, all the practitioners and representatives of the swine industry of this zone were convened to a recruitment meeting. Afterwards, twice a year the participants were convened to a meeting for promoting their continuous participation. The veterinarians that participated worked for large integrated companies as well as small individual farms. Several meetings with representatives of the swine sector and veterinary practitioners took place to define and agree what data fields to include in the app, which could be executed by desktop computer, smartphone, tablet, or other mobile device. Data from farms was supplemented with diagnostic test results if samples had been submitted to the official laboratory. A program was created to analyze the data automatically and report the health status of the swine population to veterinary practitioners that participated. This app is currently accessible using a user code and password through the link: http://www.gsplleida.net/es/content/app-del-gsp. Figure 1 shows the app interface with the fields to be filled out by a user detecting a clinical outbreak in a pig farm.

### Data Source, Types and Preparation

Data were mainly sourced from veterinarians who routinely visited the pig farms. If a veterinarian detected pigs with clinical signs during a visit to a farm, he/she registered the following variables in the app for each clinical event at farm level: severity of the clinical event, date of the visit, official identification of the farm, company to which the farm belonged, location of the farm, type of animal (i.e., sows or pigs), category of age affected, body system affected, lesions observed during necropsy (if applicable), vaccines applied, and presumptive diagnosis of the disease. The veterinarian identified the affected body system according to the clinical signs observed in swine, distinguishing between respiratory, digestive, neurological, locomotor, dermatological, and reproductive system. In the event of detecting multiple disorders in the same farm (e.g., respiratory and digestive), each sign could be recorded individually. Moreover, the veterinarian indicated the most plausible presumptive diagnosis based on his/her clinical experience. The presumptive diagnoses comprised a closed list of endemic diseases that included: porcine pleuropneumonia (APP- due to *Actinobacillus pleuropneumoniae*), porcine circovirus associated disease (due to Porcine Circovirus type 2), clostridiosis (due to *Clostridium* spp.), unspecific diarrhea (when the microorganism involved was unknown), swine dysentery (due to *Brachyspira hyodisenteriae*), colibacillosis (due to *Escherichia coli*), exudative epidermitis (due to *Staphylococcus hycus*), streptococcosis (due to *Streptococcus suis*), Glässer's disease (due to *Haemophilus parasuis*), swine influenza (due to Swine Influenza virus), ileitis (due to *Lawsonia intracellularis*), leptospirosis (due to *Leptospira* spp.), mycoplasmosis (due to *Mycoplasma hyopneumoniae*), any swine parasitosis, pasteurellosis (due to *Pasteurella multocida*), rectal prolapse, matrix prolapse, porcine respiratory reproductive syndrome (due to PRRSV), atrophic rhinitis (due to *Bordetella bronchiseptica* and/or *Pasteurella multocida*), salmonellosis (due to *Salmonella* spp.), and gastric ulcers. The app allowed reporting of several presumptive diagnoses during a single visit. However, this situation was very unusual, since the clinician usually indicated a unique presumptive diagnosis. Finally, the veterinarian also categorized the severity of a clinical event as mild, moderate or severe taking into account his/her own clinical experience and considering the rates of mortality and morbidity and the negative impact of the event on productive performance.

The second source of data was the GSP official laboratory for swine diseases. If practitioners submitted clinical samples from a reported affected herd, the laboratory carried out diagnostic testing to confirm or rule out a suspected endemic disease. The type of test used and the results obtained at farm level were then recorded to the app. Data of clinical cases and laboratory confirmation testing were integrated at farm level using a relational database built by the IT services of GSP. The elapsed time between the report of a clinical event and its laboratory confirmation ranged between 24 h and a week depending on whether the diagnosis was performed by PCR, serology or microbiology.

The third data source was the official census of the active swine farms in the regions of study (i.e., Aragon, Catalonia, and Navarra) (7). This census contained the following fields: a unique identifier of the farm, the company to which the farm belonged, the municipality, the county, the province, the number of adult sows/boars, the number of fattening pigs, the number of piglets in nursery, the type of production, and the UTM coordinates (x, y). The data registered by the veterinarians during their visits, and by the official laboratory were pre-processed and integrated with the census data in order to get a final data set that could be analyzed.

### Coverage Assessment

The initial aim of the GSP monitoring system was to gather data on clinical events occurring in any swine farm of Catalonia, Aragon and Navarra. The swine population of these three regions totals 6,741 active pig farms, 79% of which were located in Catalonia, 18% in Aragon and 3% in Navarra. Around 90 swine practitioners routinely visited these farms, the majority of which were located in 12 of the 62 counties of the regions. Half of the farms belonged to 20 integrated swine companies.

Over the study period, the implementation of the monitoring system was partial, as not all the veterinarians used the app to report clinical events when visiting the swine farms. Initially, to evaluate the coverage achieved by the GSP system, it was assessed from which counties the swine practitioners reported clinical events. This set of counties corresponded to the accessible population. Then, the coverage was also analyzed by type of production. The comparison allowed identification of those swine farms not participating in the monitoring system and inference of results solely to the participating population.

### Spatiotemporal Analyses and Modeling of Clinical Signs and Presumptive Diagnoses

Several descriptive analyses were conducted to summarize the frequencies of clinical events with different clinical signs and presumptive diagnoses, and visualize if any spatiotemporal pattern emerged from the data.

An initial exploration was carried out to describe the trend of clinical events monitored by week. The clinical signs and the respective presumptive diagnoses were grouped and summarized in tables, maps, and bar plots. The severity of clinical signs, the types of production and the age categories of the affected animals were also analyzed to characterize the subpopulations affected. The analysis was complemented by the diagnostic testing results from the laboratory.

Next, to evidence possible patterns over time and space, the number of events with different clinical signs and presumptive diagnoses were explored at low spatiotemporal granularity (i.e., by county and week). The counts of clinical signs and presumptive diagnoses reported weekly were represented with multiple surveillance time series. These series showed the pattern of each clinical sign for each one of the 33 counties included in the population of study between January 2012 and March 2018. Moreover, the cumulative counts of clinical events were mapped monthly and yearly at county level.

### Spatiotemporal Modeling Illustrated by Clinical Events of Porcine Pleuropneumonia as Presumptive Diagnosis

Counts of some clinical events grouped by clinical sign or presumptive diagnosis evidenced an overall trend and/or annual seasonality over time (e.g., clinical events such as porcine pleuropneumonia as presumptive diagnosis). To get a better understanding of the observed patterns for different groups, endemic-epidemic multivariate time series models for infectious disease counts were used (8–13). This approach considers that the incidence reported over time can be additively decomposed into two components: an endemic component (or baseline rate of cases with a stable temporal trend) and an epidemic (or autoregressive component). The endemic component includes several terms to represent the reference number of cases as the intercept, the trend and the possible seasonal variation over time. Added to these parameters, these endemic-epidemic multivariate time series models also allow the inclusion of a neighbor-driven component and random effects to explain their influence on the clinical events.

A basic formulation of the endemic-epidemic multivariate time series models can be expressed as:

(1) $$\begin{array}{l} \mu_{it}=e_{i}\upsilon_{t}+\lambda Y_{i,t-1}+\varphi \sum_{j\neq i}\omega_{ji}Y_{j,t-1} \end{array}$$

(2) $$\log(\upsilon_{t})=\alpha^{\left(\upsilon \right)}+\beta_{t}t+\gamma \sin(\varpi t)+\delta \cos(\varpi t)$$

where the mean incidence of clinical events in each county *i* at week *t* (μ_*it*_) depends on two components.

(1) An endemic component (υ_*t*_) multiplied by an offset that corresponds to the accessible population fraction located in each county (*e*_*i*_*)*. Here, υ_*t*_ is incorporated as log-linear predictor that includes an overall trend β_*t*_ and sine-cosine terms to represent an annual seasonal variation with a wave frequency ω = 2π*/*52.
(2) An epidemic component split into two parts: an autoregressive part that reproduces the incidence within county *i* (λ*Y*_*i,t* − 1_), and neighborhood effects that represent the transmission from other adjacent counties $j\left(\varphi \sum_{j\neq i}\omega_{ji}Y_{j,t-1}\right)$. These epidemic parameters λ = exp(α^(λ)^) and φ = exp(α^(φ)^) are assumed homogeneous across geographical units and constant over time.

The multivariate count time series defined at different spatiotemporal units can be fitted to a Poisson model, or a negative binomial model if we need to account for overdispersion. In the case of a negative binomial model, the conditional mean (μ_*it*_*)* remains the same, but the conditional variance increases to μ_*it*_(1+ μ_*it*_ψ_*i*_) with additional unknown overdispersion parameter ψ_*i*_ > 0.

These models are very flexible and allow the inclusion of covariates, estimated transmission weights, and random effects to eventually account for unobserved heterogeneity of the units.

In this study, to model the spatiotemporal patterns of clinical events of porcine pleuropneumonia as presumptive diagnosis, different models were evaluated adding diverse sequential extensions. Initially, a basic model was evaluated accounting for endemic and epidemic parameters with annual seasonality variation and overall trend. Then, other covariates, such as county neighborhood effect or population fraction of each county, were tested on the endemic or epidemic parameters, and finally random effects were tested to eventually account for unobserved heterogeneity of counties.

The most appropriate model was selected by comparing the values obtained from the Akaike Information Criterion and choosing the lowest one (14, 15).

### Reporting Information in Near Real Time

The GSP application allowed not only recording and integration of data on clinical events, but also immediate feedback to veterinary practitioners on trends and spatiotemporal evolution of events at county and regional level.

In addition, reproducible documents were created in “pdf” format to report the updated information extracted from the analyses of data. All the stakeholders and veterinarians who collaborated in the network received these reports with detailed results related to reported clinical events and spatial and temporal evolution of patterns of clinical signs and presumptive diagnoses.

It is important to notice that these reports did not show raw information. In order to protect the privacy of the participating stakeholders, the information was summarized by region or county without giving exact details on individual farms.

### Software Used for the Development of the Web Application and the Implementation of Analyses

The application of GSP was developed using the following software: HTML5 (16), Java script (17), and Angular 2 (18), and the working environment of IONIC 4 (https://ionicframework.com).

The analyses of this study were carried out using the statistical software R (19) jointly with RStudio as a development environment (20). The plotting of multiple time series, the mapping and the modeling were performed using the “surveillance” package. This package has been broadly used to monitor public health data in diverse European institutions (16). In addition, other R packages were used to make calculations and graphs: “doBy” (21), “gdata” (22), “ggplot2” (23), “lattice” (24), “psych” (25), “maptools” (26), “rgeos” (27), “foreign” (28), “plotrix” (29), “sp” (30), “rgdal”(31), and “spdep” (32).

The reproducible reports were built in Latex format (33) and compiled with RStudio (20).

## Results

### Coverage Assessment

**Figure 2:**
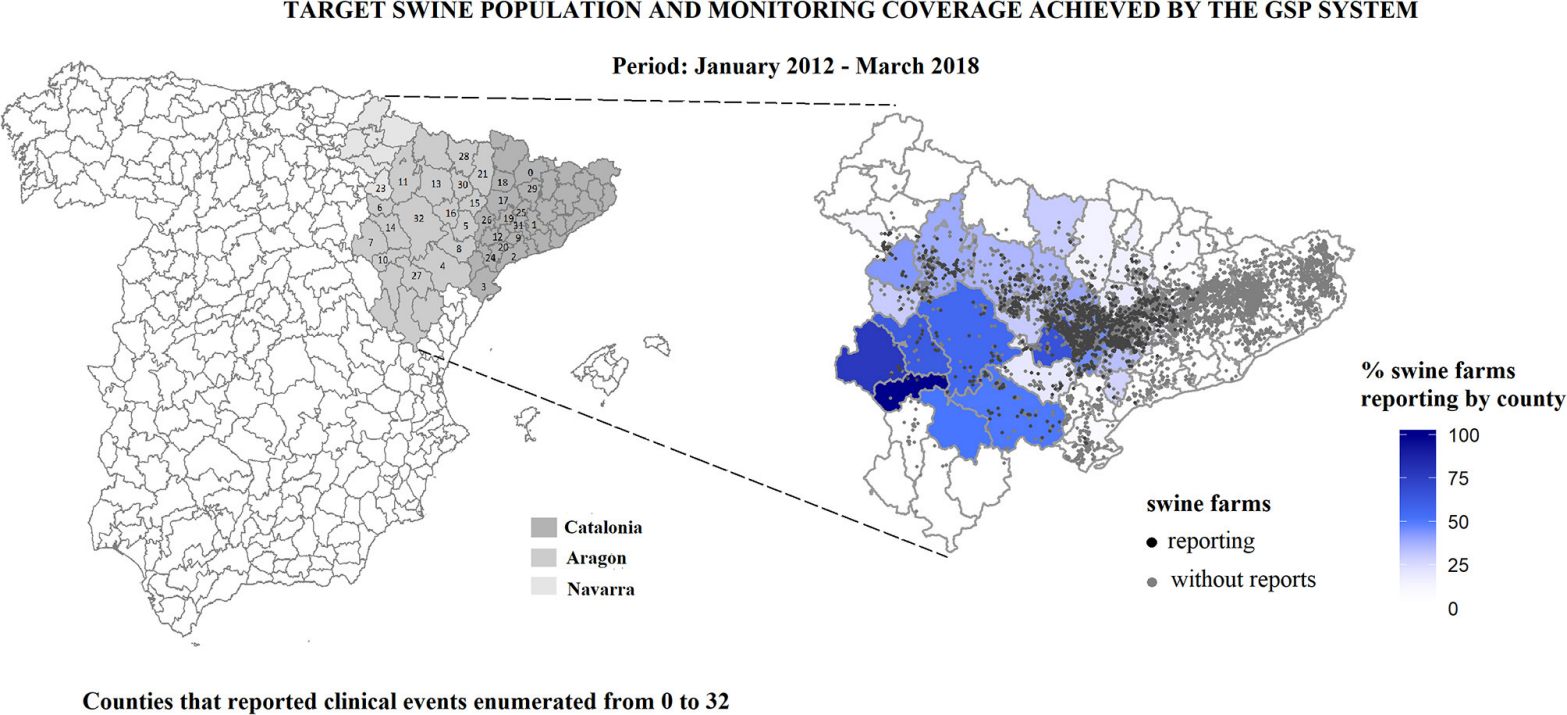
Location of the target swine population and the coverage achieved by the GSP system by county between January, 2012 and March, 2018 in Aragon, Catalonia and Navarra.

Between January 2012 and March 2018, a total of 55 practitioners out of 90 volunteered to report clinical events (i.e., veterinary participation of 61%). These practitioners covered 33 counties of the 62 existing counties and reported a median of 5 clinical events by farm with a range between 1 and 136. The 33 accessible counties comprised 4,207 swine farms, which represented 62% of 6,741 farms of the target swine population. The counties of Western Catalonia and Aragon were the most represented. Over this period, 10,654 clinical events were reported from 1,693 farms (i.e., 25 and 40% of target and accessible swine population, respectively). Figure 2 shows the location of the target swine population and the coverage achieved by the GSP system by region and county. Appendix lists the numerical county codes with their corresponding names.

Most of the clinical events were reported in fattening farms (88%), followed by sow farms and farrow-to-finish farms with 8 and 4%, respectively. The composition of the swine target population was slightly different and comprised 11 production types in which the fattening farms were the most abundant (69%), followed by sow farms (10%), and farrow-to-finish farms (10%). Moreover, it is interesting to remark that the median size of farms that reported clinical events were larger than in the target population, mainly in fattening, continuous flow finisher, and sow farms. This demonstrates that farms from integrated large-operations with highly specialized facilities were more likely to report problems to the system.

Table 1 summarizes the coverage and number of swine farms and clinical events by production type reported by the GSP monitoring system.

**Table 1:** Coverage and number of swine farms reporting by production type and number of clinical events recorded by the GSP monitoring system between January, 2012 and March, 2018.

**Production type**	**Coverage (%)**	**No. farms in the target population (%)**	**No. farms reporting (%)**	**No. clinical events (%)**
Fattening	25	4,677 (69)	1,494 (88)	9,014 (85)
Sow farm	16	664 (10)	103 (6)	947 (9)
Farrow-to-finish	8	647 (1)	50 (3)	504 (5)
Nursery	10	253 (4)	20 (1)	45 (0.4)
Gilt development unit	6	177 (3)	10 (0.6)	107 (1)
Continuous flow finisher	4	173 (3)	6 (0.4)	24 (0.2)
Multiplication	11	85 (1)	9 (0.5)	12 (0.1)
Boar stud	0	34 (0.5)	0 (0)	0 (0)
Boar development unit	0	17 (0.2)	0 (0)	0 (0)
Continuous flow nursery	11	9 (0.1)	1 (0.1)	1 (0.01)
Others	0	5 (0.1)	0 (0)	0 (0)
Total	–	6,741 (100)	1,693 (25.1)	10,654 (100)

**Figure 3:**
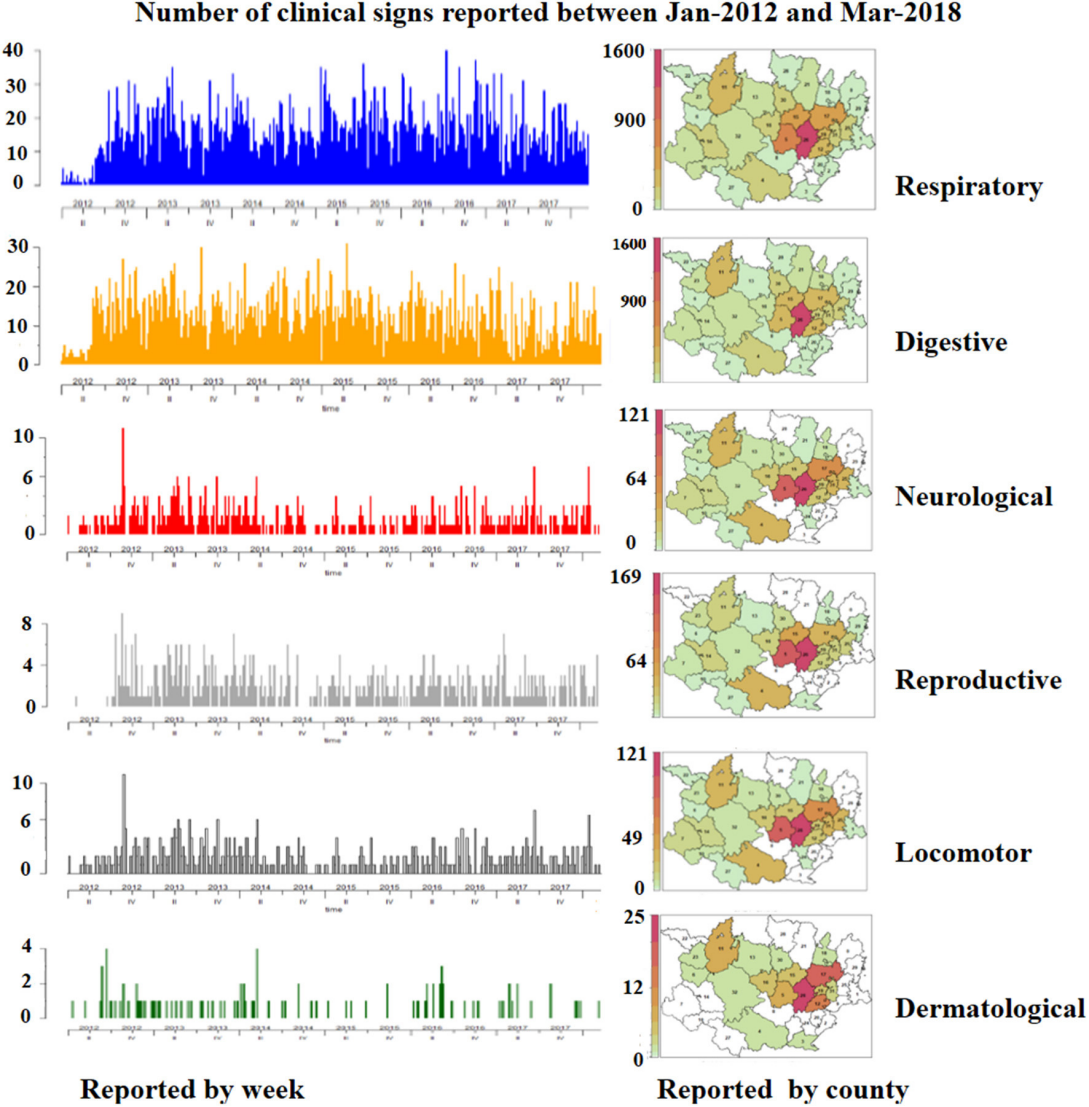
Frequency of clinical signs with their spatiotemporal distribution reported by week and by county in swine farms of Northeastern Spain between January, 2012 and March, 2018.

During the first semester of 2012 the number of reports was relatively low, but in the second semester of that year the level of reporting increased substantially being subsequently sustained throughout the whole study period (see Figure 3).

### Spatiotemporal Descriptive Analyses of Clinical Signs and Presumptive Diagnoses

From 10,654 clinical events a total of 14,971 clinical signs were reported, most of them in growing pigs (85%). In general, the degree of severity of clinical events detected in growing pigs farms (nursery and fattening pigs) was milder than in sow farms (sows and/or nursery pigs). In both growing pigs and sow farms, respiratory clinical signs were the most frequent, followed by digestive, neurological, locomotor, reproductive, and finally dermatological signs. A combination of several clinical signs was observed in 30% of these events (*n* = 3,182). The most frequent combination was digestive and neurological signs (7%), followed by respiratory and neurological (5%), respiratory and locomotor (4%), and respiratory and reproductive (4%). Figure 3 summarizes the frequency of all clinical signs reported with their respective spatiotemporal distribution by county and week.

The main presumptive diagnoses associated with respiratory clinical signs were diseases that belong to the porcine respiratory complex (such as swine influenza, PRRS, and mycoplasmosis), followed by Glasser's disease, pasteurellosis, and porcine pleuropneumonia. The most frequent presumptive diagnosis reported in clinical events with digestive signs was colibacilosis, while the most frequent suspicion for neurological signs was Glässer's disease, PRRS for reproductive signs, streptococcosis for locomotor signs, and exudative epidermitis for dermatological signs. Less than 1% of those presumptive diagnoses were confirmed by laboratory diagnosis. Table 2 summarizes the clinical signs by type of affected animals (i.e., growing pigs or sows), the number of affected farms, the degree of severity and the associated presumptive and confirmed diagnoses.

**Table 2:** Summary of clinical signs and presumptive diagnoses differentiating growing pigs and sows (period: January, 2012–March, 2018).

**Type of clinical signs**	**Type of affected animal**	**No. of affected farms**	**No. of clinical signs**	**No. of counts by week (median and range)**	**Degree of severity**	**Presumptive diagnoses**	**No. of presumptive diagnoses**	**No. of confirmed diagnoses**
Respiratory	Growing pigs 88%	1,231	5,698	17 (1–41)	75% mild,	Swine Influenza	1212	19
					23% moderate,	Mycoplasmosis	1029	3
					2% severe	Pasteurellosis	731	3
						PRRS	875	14
						Porcine pleuropneumonia	678	8
						Glässer's disease	680	1
						Rhinitis	31	0
						Porcine circovirus associated disease	6	2
	Sows 12%	118	751	4 (1–12)	1% mild,	Swine Influenza	67	2
					96% moderate,	Mycoplasmosis	12	0
					3% severe	PRRS	82	15
						Porcine pleuropneumonia	80	2
						Glässer's disease	189	1
						Rhinitis	0	0
						Porcine circovirus associated disease	1	1
Digestive	Growing pigs 87%	1,115	4,256	13 (1–31)	73% mild,	Colibacilosis	1231	7
					25% moderate,	Unspecific diarrhea	599	0
					2% severe	Clostridiosis	670	2
						Swine dysentery	440	14
						Salmonellosis	314	4
						Ileitis	137	1
						Ulcers	71	0
						Any parasitosis	33	0
						Rectal prolapse	4	0
	Sows 13%	126	662	3 (1–14)	4% mild,	Colibacilosis	315	3
					93% moderate,	Unspecific diarrhea	42	0
					3% severe	Clostridiosis	1	2
						Swine dysentery	73	12
						Salmonellosis	93	4
						Ileitis	6	3
Neurological	Growing pigs 82%	708	1,778	5 (1–16)	75% mild,	Glässer's disease	584	0
					23% moderate,	Streptococcosis	226	2
					3% severe			
	Sows 18%	32	379	3 (1–9)	98% moderate	Glässer's disease	157	0
					2% severe	Streptococcosis	217	0
Reproductive	Growing pigs 84%	328	575	2 (1–10)	92% mild,	PRRS	573	57
					5% moderate,	Porcine circovirus associated disease	0	0
					2% severe			
	Sows 16%	71	90	1 (1–3)	17% mild,	PRRS	87	57
					60% moderate,	Porcine circovirus associated disease	2	1
					23% severe	Leptospirosis	1	0
Locomotor	Growing pigs 98%	429	654	2 (1–11)	67% mild,	Streptococcosis	618	0
					32% moderate,	Glässer's disease	56	0
					1% severe			
	Sows 2%	9	11	1 (1–2)	91% moderate,	Streptococcosis	11	0
					9% severe			
Dermatological	Growing pigs 96%	95	112	1 (1–4)	83% mild,	Exudative epidermitis	77	0
					15% moderate,	PRRS	1	0
					2% severe			
	Sows 4%	5	5	1 (1–1)	60% moderate,	Exudative epidermitis	5	0
					40% severe			

**Figure 4:**
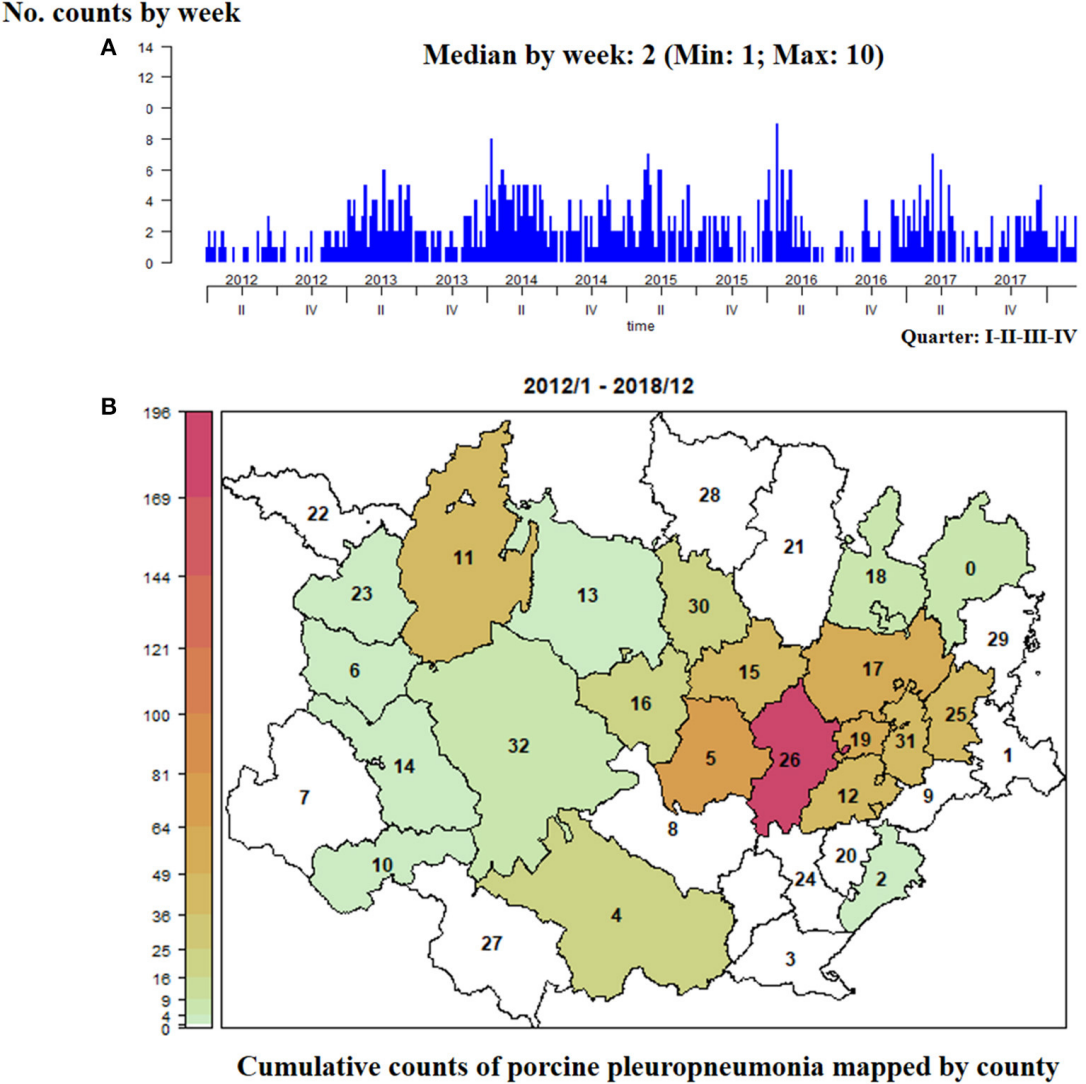
Number of clinical events with porcine pleuropneumonia as presumptive diagnosis (January, 2012–March, 2018). **(A)** Counts reported by week. **(B)** Cumulative counts mapped by county.

Next, using porcine pleuropneumonia as example of presumptive diagnosis, we illustrate how the clinical events grouped by each presumptive diagnosis were represented spatiotemporally. It is important to note that the clinical signs of porcine pleuropneumonia are quite pathognomonic, and thus clinical suspicions were a useful measure of the pattern of disease. The disease was suspected by the veterinarians on 758 occasions, most often in pigs on fattening farms (88%). Figure 4 illustrates the time series of the number of clinical events reported by week and the cumulative counts of events reported by county (see Figure 4).

**Figure 5:**
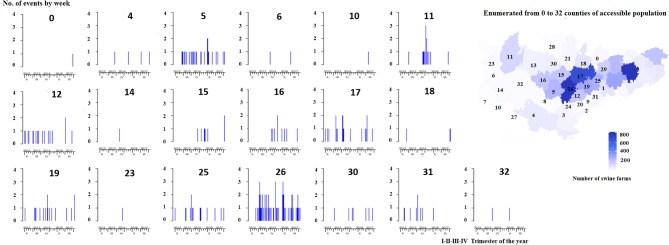
Clinical events of porcine pleuropneumonia as presumptive diagnosis reported by week and by county in Northeastern Spain between January, 2012 and March, 2018.

Moreover, using multivariate surveillance time series, trend at county level can be visually assessed and compared among counties (see Figure 5).

Figures 4, 5 show that, except for 2012, throughout the whole period of study the overall trend of reporting was quite stable with a seasonal increase each winter. From the accessible population 19 out of the 33 counties reported at least one suspicion of porcine pleuropneumonia. Although the number of weekly counts by county were relatively low (i.e., maximum 3), those counties of Western Catalonia and Aragon that had more swine farms also reported more consistently suspicions of porcine pleuropneumonia (e.g., county labeled as 26).

### Spatiotemporal Modeling for Porcine Pleuropneumonia as Presumptive Diagnosis

**Figure 6:**
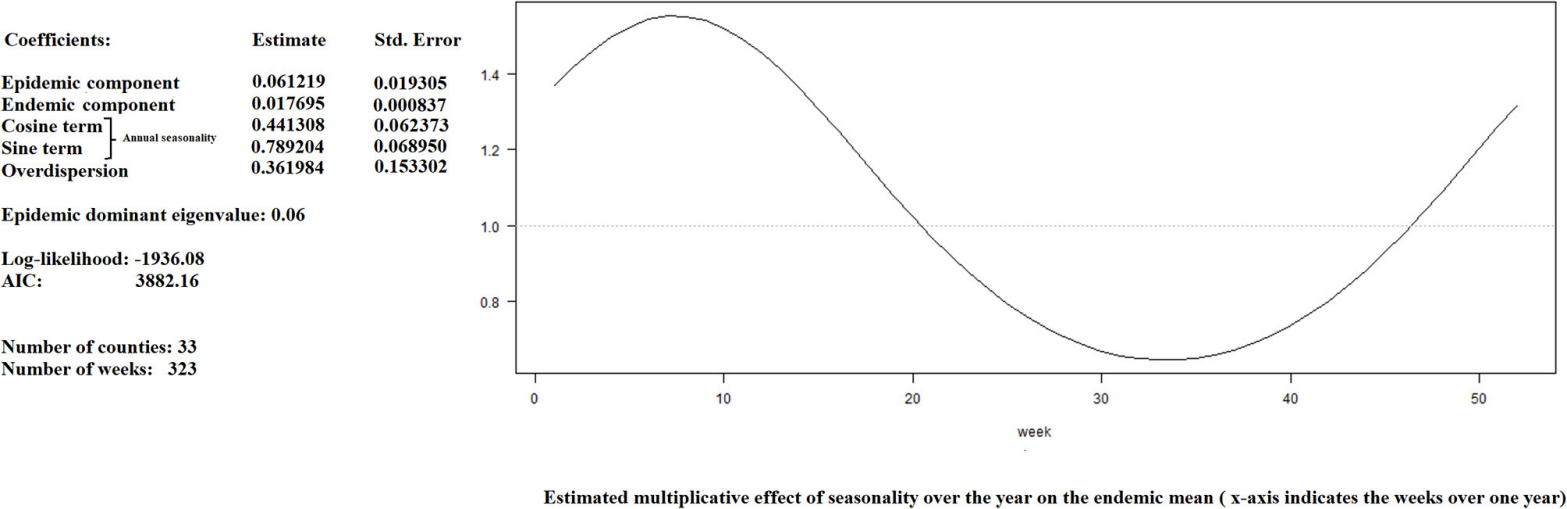
Summary of the fitted model to data of suspected clinical events of porcine pleuropneumonia recorded by the GSP system between January, 2012 and March, 2018: coefficients and resulting multiplicative effect of seasonality on the endemic component.

The data on porcine pleuropneumonia as presumptive diagnosis was fitted using a negative binomial model. This model included an endemic component with a marked seasonality that increased between January and March and an epidemic component. In this case, the influence of random effects structures could not be assessed due to the lack of convergence. The coefficients of the fitted model and the resulting multiplicative effect of seasonality on the endemic component for the suspected clinical events of swine pleuropneumonia are shown in Figure 6.

### Reporting Information in Near Real Time

**Figure 7:**
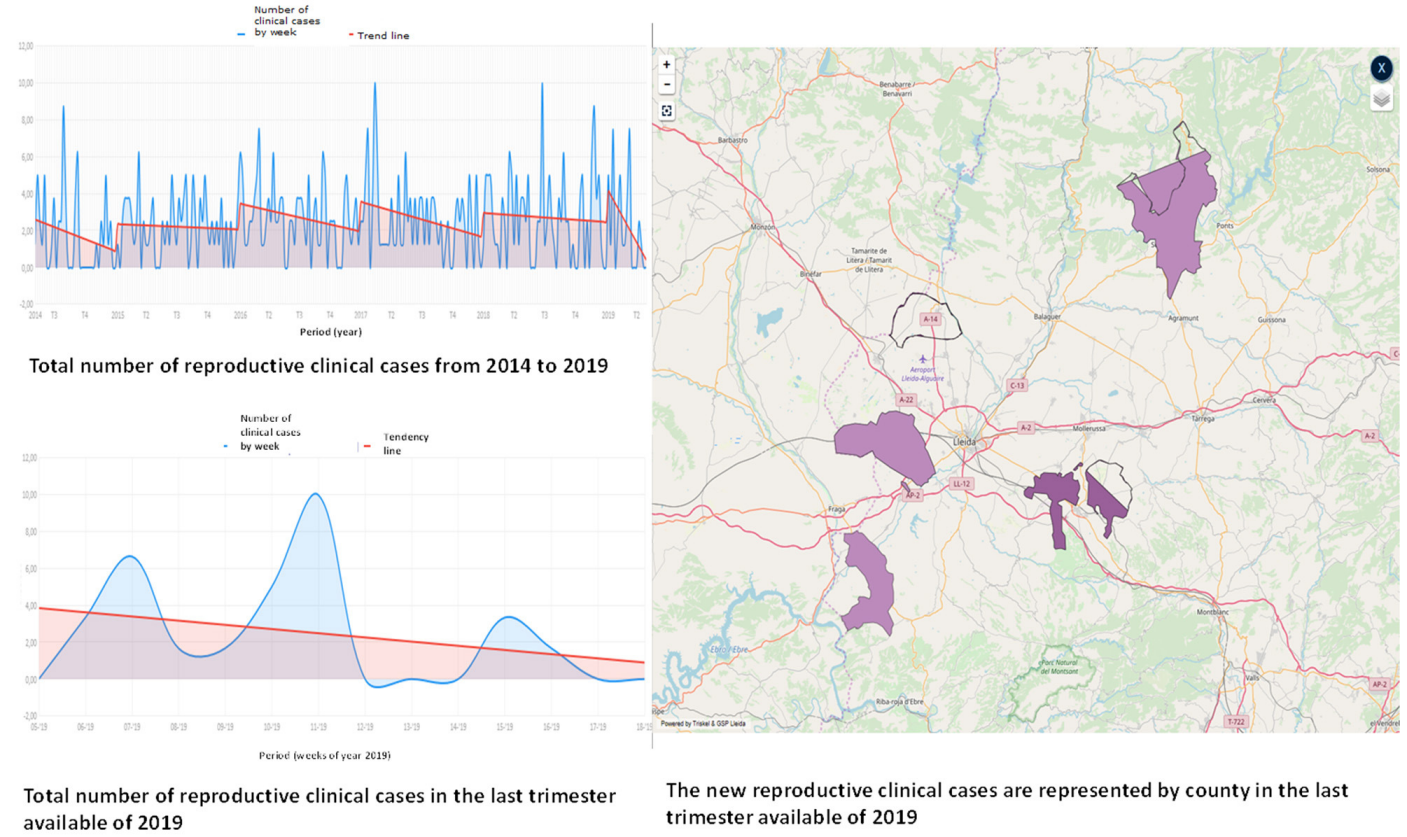
Illustration of layout of the GSP web application to visualize the area and the trend of reported reproductive clinical events.

Finally, with the aim of providing a continuous feedback to stakeholders and veterinarians, and communicate information on the health status of the population in near real time, the system produced different reports. Directly from the web application the user could get the area where the clinical events were reported during the last 3 months and the trend (see Figure 7).

Moreover, every month the stakeholders received a brief report summarizing the information of the clinical events reported by age, type of farms, and counties. Each year they also received a very detailed report of the monitoring conducted and the results of the models that evaluate the pattern of some clinical signs and presumptive diagnoses.

## Discussion

Traditionally, in Spain, the reporting of many endemic swine diseases through passive surveillance has been very disperse and scarce, and the information required for making proper decisions in health management at population level has often been poor (4). The development of new user-friendly and standardized digital methods to report and analyze data on clinical events can help to determine the frequency and the evolution of diseases at population level (5, 34). Recent initiatives have been carried out to monitor some endemic diseases in swine populations using these methods. Although many authors have pointed out the potential for near real-time monitoring, system development faces several technical, social and communication challenges in order to define data standards and data-sharing agreements (1–3, 5). Implementation involves a multidisciplinary approach and the participation of the swine sector. This was the case with the GSP system, built in collaboration with researchers in swine diseases, lab technicians, computer scientists, practitioners, epidemiologists and with the support of producers of Northeastern Spain, all of whom were fully committed to the initiative.

The coverage assessment of the GSP system allowed identification of the accessible population under monitoring. This study, based on data collected over more than 6 years, shows that implementation was gradual during the first year (2012), while in subsequent years the practitioners registered clinical events on a regular basis. The spatial coverage of the target swine population was partial and varied between counties (see Figure 2) and types of production (see Table 1). Despite this limitation, the monitoring system achieved the collection of data from an important proportion of fattening farms from integrated large operations in 33 counties, mainly concentrated in Western Catalonia and Aragon. In total 55 out of 90 swine practitioners in Northeastern Spain volunteered to participate. Veterinarians usually worked in a specific area and this could lead to spatiotemporal clustering of reporting of clinical events. In each county, the clinical events were reported by veterinarians from different companies so, to ensure standardized reporting, the same training was provided to all the system participants.

To improve the coverage in areas where the app was not used, our suggestions are to hold more meetings explaining the benefits of the information provided by this kind of monitoring and try to sort out the problems that prevent practitioners from participating. On the other hand, since this system did not allow differentiating if a specific farm was not visited or the veterinarian did not detect any clinical event, we recommended adding a field in the application to record all visits of the practitioner, even when no disease was observed. We believe that the recording of all the clinical inspections carried out would help improve the assessment of the coverage of the system and serve to demonstrate the absence of endemic diseases.

The spatiotemporal descriptive analyses from clinical events reported at farm level allowed the identification in near real-time of the most frequent clinical signs and presumptive suspicions at county and regional level. This easily accessible and current information could be useful to veterinary clinicians and stakeholders for decision making. For example, if a practitioner knew that the incidence of an endemic disease had increased in neighboring farms, he/she could decide to implement or modify preventive measures (e.g., vaccination) or take samples in other swine farms to confirm the presence or absence of infection.

In addition, the spatiotemporal descriptive analyses of retrospective data from clinical events allowed assessment of the evolution of different clinical signs and endemic diseases, comparison of different subpopulations and identification of groups of farms, areas, or periods with higher incidence of specific problems. This long-term monitoring could help to determine the baseline frequency of clinical signs or endemic diseases, assess the influence of different factors on disease presence, and predict clinical events. In our study, the results of these analyses showed that the most frequent clinical signs reported from the accessible population were respiratory, followed by digestive and neurological. Moreover, as example of a more detailed analysis, by combining data of clinical signs and presumptive diagnoses, we observed that the practitioners mainly associated these signs with diseases of the respiratory complex (such as swine influenza, mycoplasmosis, or PRRS), followed by pasteurellosis, porcine pleuropneumonia, and Glässer's disease.

However, it is important to note that due to the small number of samples (<1% of events), most of these presumptive diagnoses were not confirmed by the laboratory. The main reason why swine practitioners did not take samples was that they believed that the laboratory confirmation would not change the medical interventions to undertake at farm level; and thus, they preferred to avoid extra-costs and logistical difficulties. The monitoring of presumptive diagnoses without laboratory confirmation could result in false alerts being raised. To minimize this limitation, we suggest identifying subpopulations frequently affected by clinical signs or endemic disease suspicions, communicating the information to practitioners and recommending submission of samples for laboratory confirmation. Furthermore, the reporting of presumptive diagnoses was defined as a closed list of possible endemic diseases and the option to report other endemic diseases (not included in the list) or exotic diseases was not considered. To improve this reporting, we suggest including the option of “other suspicion” within an open field, where the veterinarian could record other diagnoses or findings.

The reproducible reports created by the GSP system provided updated and continuous information to the practitioners and swine stakeholders who participated. These reports showed visually the frequency of health problems at county and regional level, allowed the identification of their spatial distribution and progress, and helped the decision-making of where and when actions were necessary. Practitioners and swine stakeholders benefited from sharing information of clinical events occurring in the neighboring areas to plan control measures against these infections at farm level. Moreover, this system facilitated communication within the swine sector in Northeastern Spain and promoted co-operation. However, at this initial stage, the interventions to undertake in the event of alert at population level had not been agreed upon by different practitioners and private stakeholders, so the system was not ready to be used to plan specific actions. A future potential use of this system would be as surveillance system in order to detect outbreaks or aberrations. Nevertheless, for directing effective control actions, we still need to gradually build more trust in the current monitoring and achieve a better consensus and commitment from the whole swine sector.

## Conclusions

Overall, we believe that the kind of monitoring system described in this study provides very useful information to detect and monitor the trend of the most frequent endemic diseases, identify specific health problems and to enhance communication within the swine sector. A consensual and broad implementation of the system on the whole target population could shorten the response time to prevent and control certain diseases, decreasing productive, and sanitary losses.

Further research could be directed at identifying disease characteristics and modeling other covariates of interest at company or county level to further benefit endemic disease control within the swine industry.

## Data Availability Statement

The datasets generated for this study are available on request to the corresponding author.

## Ethics Statement

The approval of our study was not required as per the local legislation. This research did not involve any specific clinical study using animal experimentation. This work has been written in agreement with the veterinary clinicians and stakeholders who provided data. All data have been analyzed in aggregate form guaranteeing their privacy and security.

## Author Contributions

AA-C conceived the research, performed the statistical analyses, developed the reproducible reports, and wrote the article. EA conceived the research, designed the app, obtained the data, and provided the layout to show the results in the app. VT and JB conceived the research, built, coordinated, and motivated the network of veterinary clinicians that participated in the GSP system. EN conceived the research, performed the laboratory tests, and interpreted the results. SN interpreted the laboratory test results and wrote the article. LF conceived the research, reviewed the statistical analyses and reproducible reports, guided the study, and wrote the article.

### Conflict of Interest

The authors declare that the research was conducted in the absence of any commercial or financial relationships that could be construed as a potential conflict of interest.

## Abbreviations

GSP: Porcine Sanitation Group
app: web application
REGA: National official farm identifier
APP: *Actinobacillus pleuropneumoniae* infection or porcine pleuropneumonia
PRRS: porcine respiratory reproductive syndrome.

## Appendix

**Table A1:** List of numerical county codes and corresponding names covered by the GSP monitoring system.

**Codes**	**County names**
0	ALT URGELL
1	ANOIA
2	BAIX CAMP
3	BAIX EBRE
4	BAJO ARAGON
5	BAJO CINCA
6	BORJA
7	CALATAYUD
8	CASPE
9	CONCA DE BARBERA
10	DAROCA
11	EGEA DE LOS CABALLEROS
12	GARRIGUES
13	HOYA DE HUESCA
14	LA ALMUNIA DE DONA GODINA
15	LA LITERA
16	MONEGROS
17	NOGUERA
18	PALLARS JUSSA
19	PLA D'URGELL
20	PRIORAT
21	RIBARGORZA
22	RIBERA ALTA ARAGON
23	RIBERA BAJA
24	RIBERA D'EBRE
25	SEGARRA
26	SEGRIA
27	SERRANIA DE MONTALBAN
28	SOBRARBE
29	SOLSONES
30	SOMONTANO
31	URGELL
32	ZARAGOZA
